# Gold wrist-assisted PFNA reduces internal complications and enhances recovery in obese osteoporotic patients with intertrochanteric femur fractures

**DOI:** 10.1371/journal.pone.0348432

**Published:** 2026-07-02

**Authors:** Zhonghan Wu, Jianyang Li, Shitan Mao, Jingtao Lu, Yao Zhao, Shuisheng Yu, Dasheng Tian, Juehua Jing, Rongguang Ao, Xinzhong Xu

**Affiliations:** 1 Department of Orthopaedics, The Second Affiliated Hospital of Anhui Medical University, Hefei, China; 2 Institute of Orthopaedics, Research Center for Translational Medicine, The Second Affiliated Hospital of Anhui Medical University, Hefei, China; 3 Department of Orthopaedics, Shanghai Seventh People’s Hospital, Shanghai, China; Southern Medical University Nanfang Hospital, CHINA

## Abstract

**Introduction:**

Obese, osteoporotic patients with intertrochanteric fractures face higher surgical risks and delayed recovery. The “Gold Wrist,” an auxiliary curved guiding instrument for proximal femoral nail anti-rotation (PFNA), was developed to assist guidewire placement and improve procedural efficiency.

**Methods:**

In this retrospective cohort study, 500 patients (almost all with BMI > 28 and varying degrees of osteoporosis) underwent PFNA using either the Gold Wrist (n = 250) or conventional instruments (n = 250). Outcomes included operative time, fluoroscopy exposure, incision length, inflammatory markers, functional recovery, internal medicine complications, mechanical complications, hospitalization cost, and estimated carbon footprint. Finite element analysis assessed device mechanical stability.

**Results:**

Finite element analysis confirmed reliable mechanical performance. The Gold Wrist group had shorter operative time (114.7 ± 34.6 vs. 127.3 ± 34.3 min), smaller incisions (6.15 ± 1.88 vs. 7.27 ± 1.79 cm), and lower TNF-α, IL-6, CRP, and CK levels (all P < 0.0001). Functional recovery was faster, with higher Harris Hip Scores (87.7 ± 2.7 vs. 84.7 ± 3.4) and greater hip motion. Internal medicine complications—including infection, deep vein thrombosis, pulmonary embolism, and cardiovascular events—were significantly reduced. Mechanical complications, including implant failure, were reported separately. Hospitalization costs and estimated carbon footprint were also lower.

**Conclusion:**

The auxiliary Gold Wrist device improves surgical precision, accelerates recovery, and reduces internal medicine complications in obese, osteoporotic patients undergoing PFNA. It offers a clinically effective and sustainable approach to managing high-risk intertrochanteric fractures.

## Introduction

Intertrochanteric fractures are among the most common injuries in the elderly population, particularly those with underlying osteoporosis [1–3]. With the global population aging rapidly, the annual incidence of hip fractures is projected to exceed 3 million cases by 2050 [2–6]. These injuries are associated with considerable morbidity, mortality, and healthcare costs [7,8], imposing a significant socioeconomic burden on individuals and health systems alike [9,10]. Timely and stable surgical fixation is essential for restoring mobility, reducing complication rates, and improving long-term outcomes [2,3,6].

Intramedullary fixation, especially using the proximal femoral nail anti-rotation (PFNA) system, has become the gold standard for treating intertrochanteric fractures due to its minimally invasive nature and superior biomechanical stability [11–14]. However, the technical success of PFNA is highly dependent on precise guidewire placement at the apex of the greater trochanter-an essential step for achieving optimal nail alignment and effective fracture reduction [15,16].

Achieving an accurate entry point can be particularly challenging in patients with high body mass index (BMI), robust musculature, or anatomical variability [11–14]. In such cases, the conventional method of guidewire insertion using a straight guiding instrument under fluoroscopy often requires multiple adjustments, leading to prolonged operative time, increased radiation exposure, and a heightened risk of complications such as cortical perforation or iatrogenic vascular injury [11–15,17].

To address these challenges, our team developed a modified guiding instrument-termed the “Gold Wrist.” Adapted from the traditional “gold finger” device, the Gold Wrist features a curved ergonomic handle that enhances maneuverability and trajectory alignment in patients with complex soft tissue anatomy. This design facilitates more accurate guidewire placement, potentially reducing fluoroscopic dependence and improving procedural efficiency and safety.

With increasing attention to efficiency, biological safety, and sustainable resource use in surgical practice, there is growing interest in innovations that bridge engineering design with clinical outcomes. This study evaluates the “Gold Wrist” in PFNA procedures by integrating finite element validation of its biomechanical stability, clinical assessment of surgical efficiency, and biological analysis of perioperative inflammatory markers. We hypothesize that its use can not only shorten operative time and reduce radiation exposure but also attenuate systemic inflammatory responses, accelerate functional recovery, and decrease the environmental footprint of orthopedic surgery. Such a multidisciplinary approach highlights the Gold Wrist as a potential model for advancing both patient-centered care and sustainable surgical innovation. A graphical summary illustrating the innovative design concept of the Gold Wrist and its associated clinical and socioeconomic benefits is provided in S1 Fig.

## Materials and methods

### Study design and setting

This retrospective cohort study was conducted at the Department of Orthopedic Trauma, Second Affiliated Hospital of Anhui Medical University. The research was officially approved by the Ethics Committee of Anhui Medical University (SL-YX2022–137). The study spanned from October 2022 to October 2023 and involved the analysis of clinical records, operative data, and follow-up outcomes. Ethical approval was obtained from the institutional review board, and informed consent was secured from all patients.

### Finite element analysis of the gold wrist instrument

A finite element model of the Gold Wrist guiding instrument was established using the imported 3D geometry (model.step). The device material was defined as 304 stainless steel with a Young’s modulus of 193 GPa and a Poisson’s ratio of 0.31, and its fatigue properties were characterized using the corresponding S–N curve. Mesh generation was performed with a global element size of 2 mm for the handle and 1 mm for the other components, with additional refinement at the openings. Surface A represented the constrained region of the instrument during surgical manipulation, whereas surface B represented the force-bearing region of the handle. Boundary conditions were applied by fixing surface A in all translational directions (X, Y, Z), while a concentrated load of 45 N was applied at surface B. The 45 N load was used as a representative manual operating force to simulate surgeon-applied handling during intraoperative use and to evaluate the deformation, stress distribution, fatigue life, and overall mechanical stability of the device under simulated clinical loading conditions.

### Patient selection

A total of 500 patients with radiologically confirmed intertrochanteric fractures were included in this retrospective cohort study. The inclusion criteria were as follows: (1) age > 60 years; (2) closed intertrochanteric fracture confirmed by X-ray and CT; (3) treatment with PFNA; (4) ability to comply with postoperative rehabilitation protocols; (5) obese status, defined as a body mass index (BMI) > 28; and (6) osteoporosis, defined according to bone mineral density (BMD) criteria as a T-score ≤ −2.5 at the relevant skeletal site, or by a documented preoperative diagnosis of osteoporosis. The exclusion criteria were: (1) pathological fractures; (2) open fractures; (3) multiple fractures or polytrauma; (4) previous ipsilateral hip surgery; (5) severe systemic illness; and (6) inability to tolerate anesthesia.

### Group allocation

Patients were divided into two equal groups: the modified group (n = 250), in which the Gold Wrist instrument was used to guide entry point creation, and the control group (n = 250), where conventional instrumentation was employed. Both groups were operated on by the same experienced surgical team to minimize variability.

### Surgical technique

All procedures were performed under general anesthesia with patients placed supine on a traction table. After closed reduction under fluoroscopic guidance, a longitudinal incision of 4–5 cm was made proximal to the tip of the greater trochanter. In the modified group, the Gold Wrist was utilized to insert the guidewire with precise alignment along the medullary canal. Standard PFNA insertion, distal locking, and wound closure followed. In the control group, a traditional straight instrument was used for guidewire placement. Intraoperative variables such as incision length, operative time, and fluoroscopy use were meticulously documented.

### Postoperative management

Both groups received identical postoperative care, including prophylactic antibiotics, anticoagulation, early mobilization, and physical therapy. Clinical and radiological follow-up assessments were conducted at 1, 3, and 6 months postoperatively.

### Outcome measures

Baseline demographic and clinical data including age, sex, comorbidities (e.g., hypertension, diabetes mellitus, coronary artery disease), and time from injury to surgery were recorded. Patients with multiple fractures, previous hip surgeries, or pathological fractures were excluded. Radiographic evaluations were conducted preoperatively and postoperatively using standard anteroposterior and lateral X-rays.

Intraoperative parameters recorded included operating time, C-arm fluoroscopy frequency and duration, incision length, pre- and postoperative hemoglobin levels, tip-apex distance (TAD), and intraoperative complications. Blood samples were collected 24 hours postoperatively to measure serum levels of TNF-α (pg/mL), IL-6 (pg/mL), CRP (mg/L), and CK (U/L). Sustainability-relevant metrics such as the Length of hospital stay, Physical therapy time, and instrument repositioning events were also tracked. Fluoroscopy procedures were timed to the second using digital timestamps.

Postoperative recovery metrics included time to partial and full weight-bearing, total hospital stay, number and duration of rehabilitation sessions, final Harris Hip Scores (HHS), and range of motion at six-month follow-up. Additionally, environmental and economic indicators were evaluated. Hospitalization cost was calculated based on itemized charges retrieved from electronic medical records. Carbon footprint estimates per case were derived using standard conversion factors correlating electricity use, consumables, and rehabilitation resource consumption [18–21]. The carbon footprint per surgical case was estimated based on the methodology reported by The Journal of Bone & Joint Surgery (2024), which assessed orthopedic procedures such as hip and knee operations [22]. According to the report, the average carbon emissions per procedure ranged from 53.5 to 125.9 kg CO₂-equivalent, with energy use accounting for 57.5% and supply chain-related emissions for 34.6% of the total [22]. Given the similar surgical setup and resource utilization, we applied this framework to our intramedullary nailing procedures and derived case-level estimates accordingly. Training time for community-level surgeons was also assessed to evaluate the practicality and adaptability of the Gold Wrist.

### Ethics approval

Approved by the Ethics Committee of Anhui Medical University (SL-YX2022–137). All participants provided written informed consent.

### Statistical analysis

All statistical analyses were performed using SPSS version 24.0. Continuous variables were expressed as mean ± Std. Error of Mean (SEM) and compared using Student’s t-test or Mann-Whitney U-test as appropriate. Categorical variables were compared using Chi-square or Fisher’s exact test. Ordinal data were analyzed using the rank-sum test. A p-value of less than 0.05 was considered statistically significant.

## Results

### Device introduction and finite element analysis results

**Fig 1:**
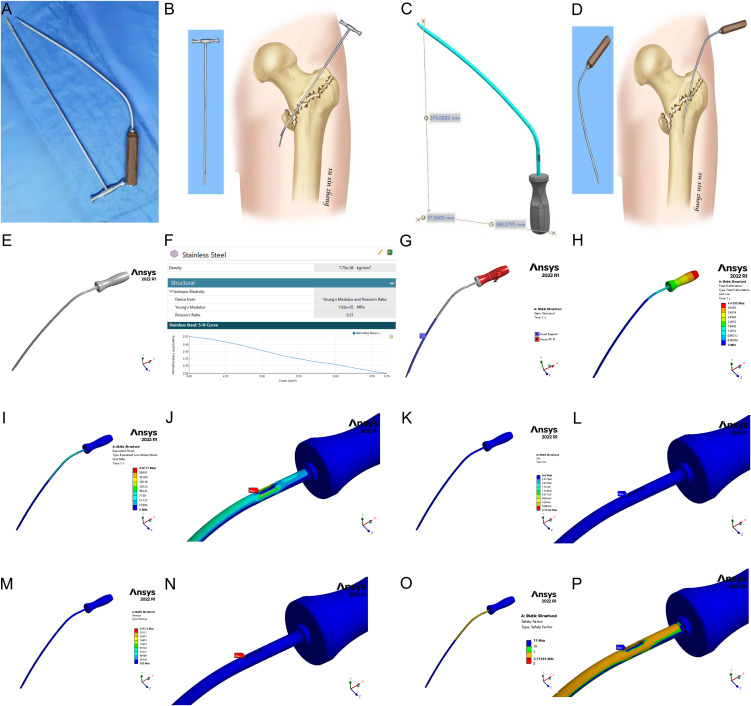
Overview and Finite element analysis of the Gold Wrist guiding instrument. **(A)** Photograph showing the traditional “Gold Finger” (bottom) and the newly developed “Gold Wrist” (top). **(B)** Technical specifications of the Gold Wrist. **(C-D)** Schematic illustrations showing the use of the Gold Finger (C) and Gold Wrist (D) in entry point creation during intramedullary nailing fixation for intertrochanteric fractures. The Gold Finger has a higher risk of breaching the medial cortex of the femur, whereas the Gold Wrist provides more accurate guidance and minimizes the risk of cortical perforation. **(E)** 3D model geometry. **(F)** S-N curve of 304 stainless steel used for fatigue analysis. **(G)** Boundary condition setup, showing fixed support at surface A and a concentrated load of 45 N applied at surface **B. (H)** Total deformation distribution, with maximum displacement occurring at the free end of the handle (4.4 mm). **(I-J)** Equivalent von Mises stress distribution and local magnification, highlighting peak stress concentrated around the opening region (232.8 MPa). **(K-L)** Fatigue life prediction and local magnification, indicating sufficient service life under repeated loading cycles. **(M-N)** Damage distribution and local magnification, demonstrating no critical failure regions in the device. **(O-P)** Safety factor distribution and local magnification, confirming overall structural stability with minimum values greater than 1.

Fig 1A-D shows the relevant parameters of devices used in this study. The S–N curve indicates that the instrument will not undergo fatigue failure under actual clinical use conditions (Fig 1E-F). Finite element analysis demonstrated that the maximum deformation (4.4 mm) occurred at the free end of the handle (Fig 1G-H). The peak equivalent stress was concentrated around the opening region, reaching 232.8 MPa, which remained well below the yield strength of 304 stainless steel (Fig 1I-J). Fatigue analysis indicated that the instrument possessed adequate service life under repeated loading, with no critical failure regions identified (Fig 1K-N). The minimum safety factor was greater than 1 across the entire structure, suggesting reliable mechanical stability during clinical use (Fig 1O-P).

### Baseline characteristics

The baseline characteristics of the two groups are shown in Table 1. There were no significant differences in age, gender distribution, or comorbidities (hypertension, diabetes mellitus, stroke, and coronary artery disease) between groups (all P > 0.05). The mean time from injury to surgery was slightly shorter in the Gold Wrist group (6.47 ± 2.91 days) than in the control group (7.22 ± 3.92 days, P = 0.015).

**Table 1:** Comparison of baseline data of patients of two groups.

**Variable**	**Control Group (n = 250)**	**Gold Wrist Group (n = 250)**	**t/χ² Value**	**P Value**
**Age (years)**	74.24 ± 10.15	75.82 ± 9.24	*t* = 1.820	0.069
**Gender, n (%)**			χ2=0.081	0.777
**Male**	118 (48)	126 (28)		
**Female**	132 (52)	134 (72)		
**Comorbidities, n (%)**				
**Hypertension**	36 (14)	40 (16)	χ2=0.248	0.618
**Diabetes mellitus**	40 (16)	42 (17)	χ2=0.058	0.81
**Stroke**	22 (9)	20 (8)	χ2=0.104	0.747
**Coronary artery disease**	28 (11)	30 (12)	χ2=3.48	0.78
**Time from injury to surgery (days)**	7.22 ± 3.92	6.47 ± 2.91	*t* = 2.441	0.015

The chi-square test with continuity correction was used for hypertension and diabetes; “-” indicates Fisher’s exact test.

### Intraoperative parameters

**Fig 2:**
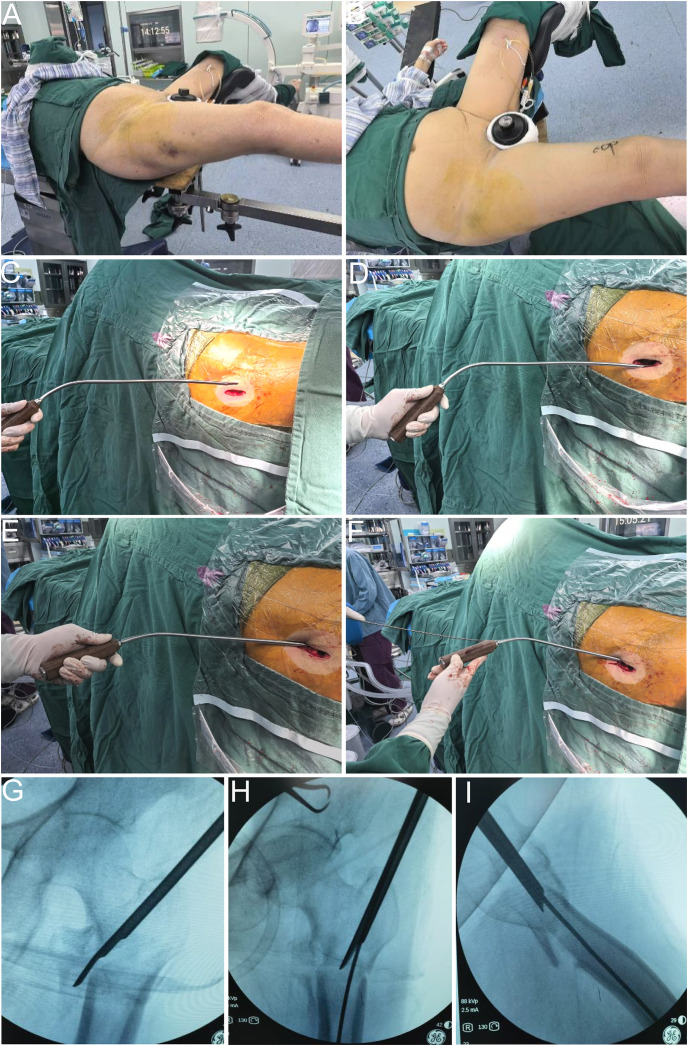
Intraoperative application of the Gold Wrist. **(A-B)** Patient positioned on a traction table. **(C-D)** Initial probing of the entry point using the Gold Wrist. **(E-F)** The Gold Wrist inserted into the distal medullary cavity to guide a long guidewire across the fracture site. **(G)** Fluoroscopic image demonstrating the tendency of the Gold Finger to breach the fractured medial femoral cortex during entry point creation. (H-I) Fluoroscopic images showing Gold Wrist successfully guided the guidewire across the fracture site while avoiding penetration of the medial cortex of the femur.

Detailed data can be found in Table 2. Compared with the control group, the Gold Wrist group had a significantly shorter mean operating time (114.7 ± 34.6 vs. 127.3 ± 34.3 min, P < 0.0001) and smaller incision length (6.15 ± 1.88 vs. 7.27 ± 1.79 cm, P < 0.0001). Postoperative hemoglobin was higher in the Gold Wrist group (90.9 ± 2.2 vs. 80.2 ± 3.0 g/L, P < 0.0001). The quality of fracture reduction was superior in the Gold Wrist group, with more patients achieving excellent reduction (90% vs. 65%, P = 0.042). Trauma- and inflammation-related biomarkers (TNF-α, IL-6, CRP, CK) were all significantly lower in the Gold Wrist group (all P < 0.0001). The tip-apex distance was slightly greater in the Gold Wrist group but remained within the safe range (18.04 ± 0.90 vs. 17.54 ± 1.02 mm, P < 0.0001). The usage of surgical instruments during the operation is shown in Fig 2.

**Table 2:** Comparison of perioperative data between the two groups.

**Variable**	**Control Group (n = 250)**	**Gold Wrist Group (n = 250)**	**t/z Value**	**P Value**
**Operating time (min)**	127.30 ± 34.28	114.70 ± 34.63	*t* = 4.096	< 0.0001
**Preoperative hemoglobin (g/L)**	108.80 ± 16.56	109.60 ± 16.92	*t* = 0.526	0.0859
**Incision length (cm)**	7.27 ± 1.79	6.15 ± 1.88	*t* = 6.829	< 0.0001
**Postoperative hemoglobin (g/L)**	80.21 ± 2.99	90.90 ± 2.23	*t* = 17.40	< 0.0001
**Quality of fracture reduction, n (%)**			*z* = −5.0	0.042
**Excellent**	162 (65)	226 (90)		
**Good**	68 (27)	20 (8)		
**Acceptable**	20 (8)	4 (2)		
**Postoperative trauma- and inflammatory biomarkers**				
**TNF-α (pg/mL)**	5.45 ± 1.57	3.91 ± 0.87	*t* = 13.64	< 0.0001
**IL-6 (pg/mL)**	17.81 ± 2.17	12.85 ± 1.54	*t* = 29.49	< 0.0001
**CRP (mg/L)**	101.70 ± 7.04	66.17 ± 4.25	*t* = 68.20	< 0.0001
**CK (UI/L)**	233.60 ± 7.70	187.00 ± 6.17	*t* = 74.75	< 0.0001
**Tip-apex distance (mm)**	17.54 ± 1.02	18.04 ± 0.90	*t* = 5.84	< 0.0001

### Postoperative recovery and follow-up

**Fig 3:**
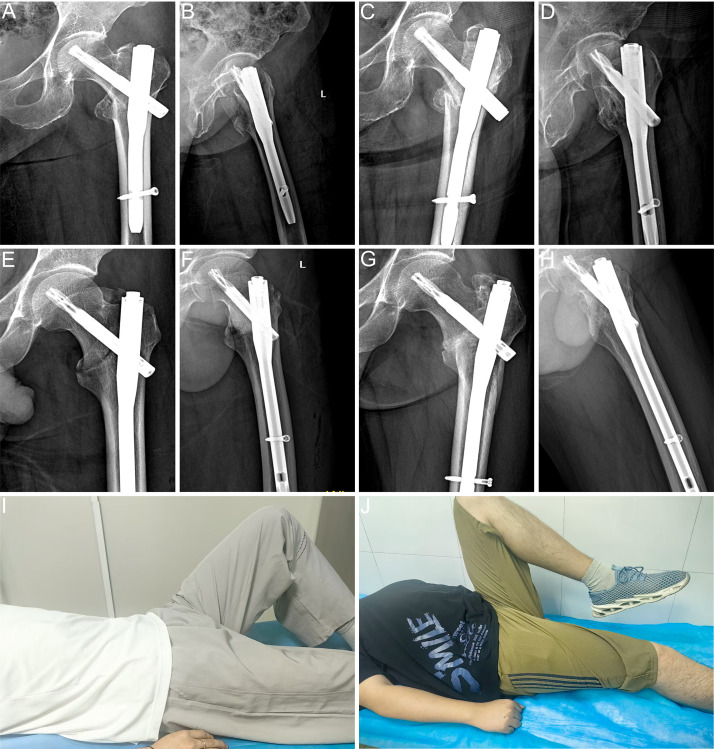
Postoperative outcomes with Gold Finger and Gold Wrist. **(A-B)** Patient 1 underwent entry point creation with the Gold Finger; fracture reduction quality was rated as “good.” **(C-D)** At 6-month follow-up, bone callus formation was observed, but the fracture line remained visible, indicating slightly delayed fracture healing. **(E-F)** Patient 2 underwent entry point creation with the Gold Wrist; fracture reduction quality was rated as “excellent.” **(G-H)** At 6-month follow-up, abundant callus formation and blurred fracture line indicated more advanced fracture healing. **(I-J)** The postoperative joint range of motion in the Gold Wrist group (J) was greater than that in the Gold Finger group **(I)**.

Detailed data can be found in Table 3. Patients in the Gold Wrist group regained mobility faster, with shorter times to partial (7.66 ± 0.89 vs. 8.98 ± 0.79 days, P < 0.0001) and full weight-bearing (12.24 ± 0.65 vs. 13.34 ± 0.78 days, P < 0.0001). Functional recovery was also better, reflected by higher final Harris Hip Scores (87.7 ± 2.7 vs. 84.7 ± 3.4, P < 0.0001) and greater hip flexion-extension range (134.8 ± 5.5° vs. 129.6 ± 6.1°, P < 0.0001). The incidence of complications was consistently lower in the Gold Wrist group. Rates of infection (1% vs. 5%, P = 0.007), deep vein thrombosis (1% vs. 6%, P < 0.0001), pulmonary embolism (1% vs. 6%, P = 0.006), stroke or myocardial infarction (1% vs. 5%, P = 0.015), and implant failure (1% vs. 4%, P = 0.030) were all significantly reduced. One-year postoperative mortality was also markedly lower in the Gold Wrist group (2% vs. 10%, P = 0.0003). The details of the patient’s fracture healing and functional recovery can be found in Fig 3.

**Table 3:** Comparison of postoperative follow-up data between the two groups.

Variable	Control Group (n = 250)	Gold Wrist Group (n = 250)	t/χ² Value	P Value
**Time to partial weight-bearing (days)**	8.98 ± 0.79	7.66 ± 0.89	*t* = 17.51	< 0.0001
**Time to full weight-bearing (days)**	13.34 ± 0.78	12.24 ± 0.65	*t* = 17.12	< 0.0001
**Final Harris Hip Score**	84.72 ± 3.36	87.68 ± 2.73	*t* = 10.81	< 0.0001
**Final hip flexion-extension range (°)**	129.60 ± 6.10	134.80 ± 5.49	*t* = 10.02	< 0.0001
**Postoperative complications n (%)**				
**Infection**	12 (5)	2 (1)	^*–*^	0.007
**Deep vein thrombosis (DVT)**	16 (6)	2 (1)	^*–*^	<0.0001
**Pulmonary embolism (PE)**	15 (6)	3 (1)	^*–*^	0.006
**Stroke/ Myocardial infarction (MI)**	13 (5)	3 (1)	^*–*^	0.015
**Implant failure**	10 (4)	2 (1)	^*–*^	0.03
**1-year postoperative mortality**	25 (10)	5 (2)	^*–*^	0.0003

The chi-square test with continuity correction was used for 1-year postoperative mortality,; “-” indicates Fisher’s exact test.

### Economic and environmental outcomes

Detailed data can be found in Table 4. The Gold Wrist group demonstrated better intraoperative and perioperative efficiency, with fewer C-arm fluoroscopy uses (13.1 ± 2.2 vs. 17.2 ± 2.4, P < 0.0001) and shorter fluoroscopy time (77.4 ± 3.2 vs. 110.0 ± 3.6 s, P < 0.0001). Length of hospital stay (12.5 ± 3.0 vs. 15.7 ± 3.1 days, P < 0.0001) and physical therapy time (9.4 ± 3.0 vs. 13.8 ± 3.0 h, P < 0.0001) were significantly shorter in the Gold Wrist group. Total hospitalization cost was lower (15,431 ± 1,139 vs. 19,307 ± 1,210 CNY, P < 0.0001), as was the estimated carbon footprint per case (62.8 ± 5.9 vs. 88.6 ± 6.5 kg CO₂-eq, P < 0.0001). Training time for community doctors was also reduced (5.5 ± 2.8 vs. 8.4 ± 3.3 h, P < 0.0001).

**Table 4:** Economic and Resource Efficiency Analysis.

Variable	Control Group (n = 250)	Gold Wrist Group (n = 250)	t Value	P Value
**C-arm fluoroscopy number**	17.24 ± 2.36	13.12 ± 2.18	*t* = 20.28	<0.0001
**C-arm fluoroscopy duration (seconds)**	110.0 ± 3.64	77.38 ± 3.19	*t* = 106.6	<0.0001
**Length of hospital stay (days)**	15.66 ± 3.06	12.50 ± 2.97	*t* = 11.70	<0.0001
**Physical therapy time (hours)**	13.78 ± 3.04	9.42 ± 2.96	*t* = 16.26	<0.0001
**Hospitalization cost (CNY)**	19307 ± 1210	15431 ± 1139	*t* = 36.89	<0.0001
**Estimated carbon footprint per case (kg CO₂-eq)**	88.62 ± 6.49	62.82 ± 5.93	*t* = 46.43	<0.0001
**Training time for community doctors (hours)**	8.44 ± 3.30	5.53 ± 2.77	*t* = 10.69	<0.0001

## Discussion

This study demonstrates that the Gold Wrist can enhance surgical efficiency and improve patient recovery in the management of intertrochanteric fractures. By enabling more accurate guidewire placement and reducing operative time, the device streamlines the procedure and minimizes unnecessary intraoperative adjustments. These improvements not only support better functional outcomes but also reflect more efficient use of operative resources [3,9,10,23,24]. In an era where healthcare systems face increasing demands for timely, cost-effective care, such innovations offer practical benefits that extend beyond the immediate surgical setting [18–21,25,26]. The finite element results, showing uniform stress distribution and high fatigue safety factors, provide a mechanical explanation for the observed intraoperative stability and reduced soft-tissue interference in clinical practice.

The Gold Wrist addresses the limitations of traditional straight-handled instruments by introducing a curved design that facilitates entry point alignment even in patients with challenging anatomy. It minimizes soft tissue interference, reduces operative time and radiation exposure, and lowers the risk of vascular injury by ensuring more precise guide wire trajectory. Finite element analysis indicated that the maximum deformation and peak stress of the device remained well below the material limits, confirming its mechanical stability under clinical loads. This study confirms the clinical advantages of the Gold Wrist in both intraoperative safety and postoperative functional recovery. In the intramedullary nail closed reduction surgery for femoral and tibial shaft fractures, “Golden Finger” is a commonly used auxiliary device, which can assist in guiding the long guide pin to smoothly pass through the fracture site and enter the distal medullary cavity, thereby achieving closed reduction of the fractures [27,28]. However, the traditional Golden Finger is of a straight tubular structure, and during the insertion process, the handle part often gets blocked by the soft tissues of the patient’s body, making it difficult to maintain parallelism with the longitudinal axis of the long bone, thereby affecting the accuracy and smoothness of the operation [16,29,30]. This study overcame the above limitations by changing the handle of the device from a straight design to a curved structure, enabling the auxiliary retractor to more easily align with the distal end of the fracture and smoothly enter the medullary cavity during the operation. Fig 1A-D shows the structural and functional comparison between the traditional Golden Finger and the modified device used in this study during actual operation. Due to the change in the shape of the device and the evolution of its usage method, the research team also underwent multiple discussions in the naming process. Eventually, the modified device was named “Golden Wrist”, which not only vividly reflects its “wrist-shaped” structure with a curve, but also continues the naming system of “Golden Finger” that uses hand structures as metaphors, enhancing the logic and recognizability of the device naming.

One of the key findings was the substantial reduction in operative time and fluoroscopy exposure, both of which are essential metrics of surgical efficiency and safety [27,28]. Reduced fluoroscopy time not only benefits the surgical team by minimizing radiation exposure but also reflects a more streamlined procedure [27,28]. Higher postoperative hemoglobin levels in the modified group further support the hypothesis that the Gold Wrist reduces intraoperative trauma. Functionally, patients treated with the Gold Wrist achieved earlier partial weight-bearing and exhibited better hip range of motion and HHS at six months. These findings suggest that the instrument not only facilitates safer surgery but also contributes to more rapid postoperative recovery. This is particularly relevant for elderly patients, for whom early mobilization is critical in preventing complications such as deep vein thrombosis, pulmonary infections, or pressure ulcers [25,26,31,32].

Relative to conventional straight guiding instruments used during PFNA, the Gold Wrist incorporates a curved trajectory-assisting design that better accommodates soft-tissue obstruction in obese osteoporotic patients and facilitates more accurate entry-point alignment. In our cohort, this design advantage translated into shorter operative time, reduced fluoroscopy use, smaller incision length, and improved early postoperative recovery. These findings extend previous reports on PFNA optimization by suggesting that a relatively simple modification of the guiding instrument may improve both intraoperative handling and short-term clinical outcomes.

Compared with previous reports, the design of the Gold Wrist addresses a common yet often overlooked challenge in orthopedic trauma surgery: navigating soft tissue resistance while preserving entry accuracy. Literature has documented various assistive tools and techniques to optimize guidewire placement, but few specifically address the limitations posed by body habitus [33,34]. Our findings complement and extend this body of knowledge by introducing a simple yet effective design modification.

Intertrochanteric fractures are a common type of fracture, and internal fixation treatment has become a consensus [33,34]. However, iatrogenic vascular injuries that may occur during internal fixation surgery are rare but have serious consequences and require high attention, with 67.03% presenting as pseudoaneurysms [35–37]. Untimely diagnosis of pseudoaneurysms may lead to serious complications, such as hematoma infection, delayed healing, compartment syndrome of bone and fascia, and deep vein thrombosis [12,37,38]. The rupture of pseudoaneurysms may cause large hematomas and even endanger life. The morphology of the fracture, such as long spiral fractures, fractures involving displacement of the lesser trochanter, and long oblique fractures extending to the femoral shaft, are common risk factors for vascular injury [35,39–41]. During surgical operations, injury to the deep femoral artery is particularly worthy of vigilance. Reasonable fracture ring compression techniques, drilling control techniques, and the selection of screw length are all key to reducing vascular injury [35,39–41]. Literature reports multiple cases of vascular injury after intertrochanteric fractures [42–46]. These cases suggest that if there are symptoms such as asymmetrical swelling on the inner side of the thigh and unreasonable drop in hemoglobin after intertrochanteric fracture surgery, one should be highly alert to the possibility of vascular injury [42–46]. Moreover, the puncture of screws or drill bits is not the only cause of vascular injury, and the tip of the small trochanteric fracture fragment may also cause vascular injury [35]. Type A fractures are particularly common with vascular injury, and the injury to the deep femoral vessels by the small trochanteric fracture fragment is particularly significant. 85% of patients will have edema, 70% will have pain symptoms, and only 1.35% will have no symptoms [35,39–41]. Therefore, special attention should be paid to the anatomical structure of the small trochanter region during surgery to avoid unnecessary damage.

The improvement in the design of the gold wrist not only enhances the efficiency and safety of the surgery, but also reduces the damage to the femoral arteries and their branch vessels to a certain extent. This is particularly important for obese or muscular patients, as the traditional opening method is more prone to cause the guide needle to deviate or protrude in these patients, resulting in vascular damage [14]. Besides a smooth opening, there are also other matters that need to be paid attention to. During the insertion of the intramedullary nail, it is necessary to drill and insert the nail within a safe area, to avoid the course of the femoral proximal vessels in the anatomy, while controlling the length of the screw and avoiding repeated operations during drilling to avoid vascular damage [46].

The Gold Wrist’s implementation led to enhanced procedural efficiency, as evidenced by reductions in fluoroscopy duration, intraoperative instrument repositioning, and overall operative time. These improvements indirectly curtail energy use and material consumption, lessening the environmental load associated with each procedure [18–21]. Furthermore, by streamlining the surgical workflow, the device contributes to reduced reliance on high-turnover resources such as sterilization supplies and disposable surgical aids [18–22]. These operational refinements suggest that device-level innovation can play a meaningful role in improving the ecological performance of routine orthopedic procedures.

Beyond environmental benefits, the introduction of the Gold Wrist also promotes economic and educational sustainability by reducing hospital resource consumption and facilitating faster skill acquisition among surgeons. From a cost-efficiency standpoint, the Gold Wrist enabled more streamlined care delivery. Shortened operative and recovery phases translated into decreased inpatient days and reduced need for rehabilitation services. In turn, this alleviated both direct expenses (e.g., bed occupancy, staffing) and indirect costs (e.g., readmission risk, extended therapy) [25,26,31]. Importantly, the reduction in surgical variability and complications supports long-term system savings by minimizing avoidable resource consumption [32]. These attributes position the device as a tool that enhances procedural value while supporting health systems’ fiscal sustainability.

In addition to operative metrics and patient-centered outcomes, the Gold Wrist may have implications for surgical education. Surgeons involved in this study observed that novice users could more quickly master entry point placement using the Gold Wrist compared to conventional tools. The ergonomic handle and improved trajectory alignment reduced the need for repeated adjustments, potentially shortening the learning curve for junior residents.

This study has several limitations. First, its retrospective and non-randomized design introduces potential selection bias and residual confounding. Second, this was a single-center study, which may limit generalizability to other institutions and patient populations. Third, although all procedures were performed by the same experienced surgical team to reduce variability, surgeon-dependent technical factors and learning-curve effects cannot be completely excluded. Fourth, follow-up duration was limited, and longer-term implant-related outcomes require further validation. Therefore, the present findings should be interpreted cautiously and confirmed in prospective multicenter studies.

## Conclusion

The Gold Wrist is a practical advancement in orthopedic surgical instrumentation that improves guidewire placement accuracy, shortens operative time, and facilitates faster patient recovery in PFNA procedures for intertrochanteric fractures. By streamlining the surgical process and reducing radiation exposure, it supports more efficient use of operative resources. With further validation in larger, multicenter studies, the Gold Wrist could be adopted as a standard adjunct, particularly for patients with challenging anatomy or higher surgical risk.

## Supporting information

S1 Fig Graphical summary of the Gold Wrist concept and its associated clinical and socioeconomic benefits compared with the conventional Gold Finger. (TIF)

## Abbreviations

BMI: body mass index
DVT: deep vein thrombosis
HHS: Harris Hip Scores
LCA: life cycle assessments
MI: myocardial infarction
PE: pulmonary embolism
PFNA: Proximal femoral nail antirotation
SEM: Std. Error of Mean
TAD: tip-apex distance
