# Correction: Gold wrist-assisted PFNA reduces internal complications and enhances recovery in obese osteoporotic patients with intertrochanteric femur fractures

**DOI:** 10.1371/journal.pone.0355219

**Published:** 2026-07-31

**Authors:** Zhonghan Wu, Jianyang Li, Shitan Mao, Jingtao Lu, Yao Zhao, Shuisheng Yu, Dasheng Tian, Juehua Jing, Rongguang Ao, Xinzhong Xu

The Study Design and Setting subsection of Materials and methods is incorrect. The correct Study Design and Setting subsection is as follows: This retrospective cohort study was conducted at the Department of Orthopedic Trauma, Second Affiliated Hospital of Anhui Medical University, the Anhui provincial regional center/leading institution of the China Trauma Rescue and Treatment Association, with responsibilities for regional referral reception, specialist outreach care coordination, and centralized postoperative follow-up management of treated patients. The study was approved by the Ethics Committee of Anhui Medical University (SL-YX2022-137). From October 2022 to October 2023, consecutive eligible patients recorded in the Department’s clinical case registry and postoperative follow-up management process, with 12-month vital-status ascertainment, were retrospectively reviewed. Deidentified clinical records, available operative-related documentation, postoperative follow-up information, and outcome data were retrieved, verified, and analyzed by the study team under the approved protocol. Informed consent was obtained from all included patients.

The Statistical analysis subsection of Materials and methods is incorrect. The correct Statistical analysis subsection is as follows: All statistical analyses were performed using SPSS version 24.0 and GraphPad Prism version 10.1.2. Continuous variables were expressed as mean ± standard deviation (SD) and compared between groups using Student’s t-test. Categorical variables were compared using the chi-square test or Fisher’s exact test, as appropriate. Ordinal variables, including fracture reduction quality, were compared using the Mann–Whitney U test. A two-sided P value of less than 0.05 was considered statistically significant.

In Tables 1, 2 and 3, there are errors in the statistical values. Please see the correct Tables 1, 2 and 3 here.

**Table 1:** Comparison of baseline data of patients of two groups.

Variable	Control Group (n = 250)	Gold Wrist Group (n = 250)	t/χ² Value	P Value
Age (years)	74.24 ± 10.15	75.82 ± 9.24	*t* = 1.820	0.069
Gender, n (%)			χ^2^=0.512	0.474
Male	118 (47)	126 (50)		
Female	132 (53)	124 (50)		
Comorbidities, n (%)				
Hypertension	36 (14)	40 (16)	χ^2^=0.248	0.618
Diabetes mellitus	40 (16)	42 (17)	χ^2^=0.058	0.81
Stroke	22 (9)	20 (8)	χ^2^=0.104	0.747
Coronary artery disease	28 (11)	30 (12)	χ^2^=0.078	0.78
Time from injury to surgery (days)	7.22 ± 3.92	6.47 ± 2.91	*t* = 2.441	0.015

**Table 2:** Comparison of perioperative data between the two groups.

Variable	Control Group (n = 250)	Gold Wrist Group (n = 250)	t/U Value	P Value
Operating time (min)	127.30 ± 34.28	114.70 ± 34.63	*t* = 4.096	< 0.0001
Incision length (cm)	7.27 ± 1.79	6.15 ± 1.88	*t* = 6.829	< 0.0001
Quality of fracture reduction, n (%)			*U* = 23186	< 0.0001
Excellent	162 (65)	226 (90)		
Good	68 (27)	20 (8)		
Acceptable	20 (8)	4 (2)		
Postoperative trauma- and inflammatory biomarkers				
TNF-α (pg/mL)	5.45 ± 1.57	3.91 ± 0.87	*t* = 13.64	< 0.0001
IL-6 (pg/mL)	17.81 ± 2.17	12.85 ± 1.54	*t* = 29.49	< 0.0001
CRP (mg/L)	101.70 ± 7.04	66.17 ± 4.25	*t* = 68.20	< 0.0001
CK (UI/L)	233.60 ± 7.70	187.00 ± 6.17	*t* = 74.75	< 0.0001
Tip-apex distance (mm)	17.54 ± 1.02	18.04 ± 0.90	*t* = 5.84	< 0.0001

**Table 3:** Comparison of postoperative follow-up data between the two groups.

Variable	Control Group (n = 250)	Gold Wrist Group (n = 250)	t/χ² Value	P Value
Time to partial weight-bearing (days)	8.98 ± 0.79	7.66 ± 0.89	*t* = 17.51	< 0.0001
Time to full weight-bearing (days)	13.34 ± 0.78	12.24 ± 0.65	*t* = 17.12	< 0.0001
Final Harris Hip Score	84.72 ± 3.36	87.68 ± 2.73	*t* = 10.81	< 0.0001
Final hip flexion-extension range (°)	129.60 ± 6.10	134.80 ± 5.49	*t* = 10.02	< 0.0001
Postoperative complications n (%)				
Infection	12 (5)	2 (1)	^*–*^	0.012
Deep vein thrombosis (DVT)	16 (6)	2 (1)	^*–*^	0.001
Pulmonary embolism (PE)	15 (6)	3 (1)	^*–*^	0.007
Stroke/ Myocardial infarction (MI)	13 (5)	3 (1)	^*–*^	0.019
Implant failure	10 (4)	2 (1)	^*–*^	0.0363
1-year postoperative mortality	25 (10)	5 (2)	χ^2^=12.80	0.0003

The chi-square test with continuity correction was used for 1-year postoperative mortality; “–” indicates Fisher’s exact test.
