# Supplementary figures and images for: Gold wrist-assisted PFNA reduces internal complications and enhances recovery in obese osteoporotic patients with intertrochanteric femur fractures
Source: PLoS One. 2026 Jul 2;21(7):e0348432. doi: 10.1371/journal.pone.0348432 (PMC13327258; doi:10.1371/journal.pone.0348432)

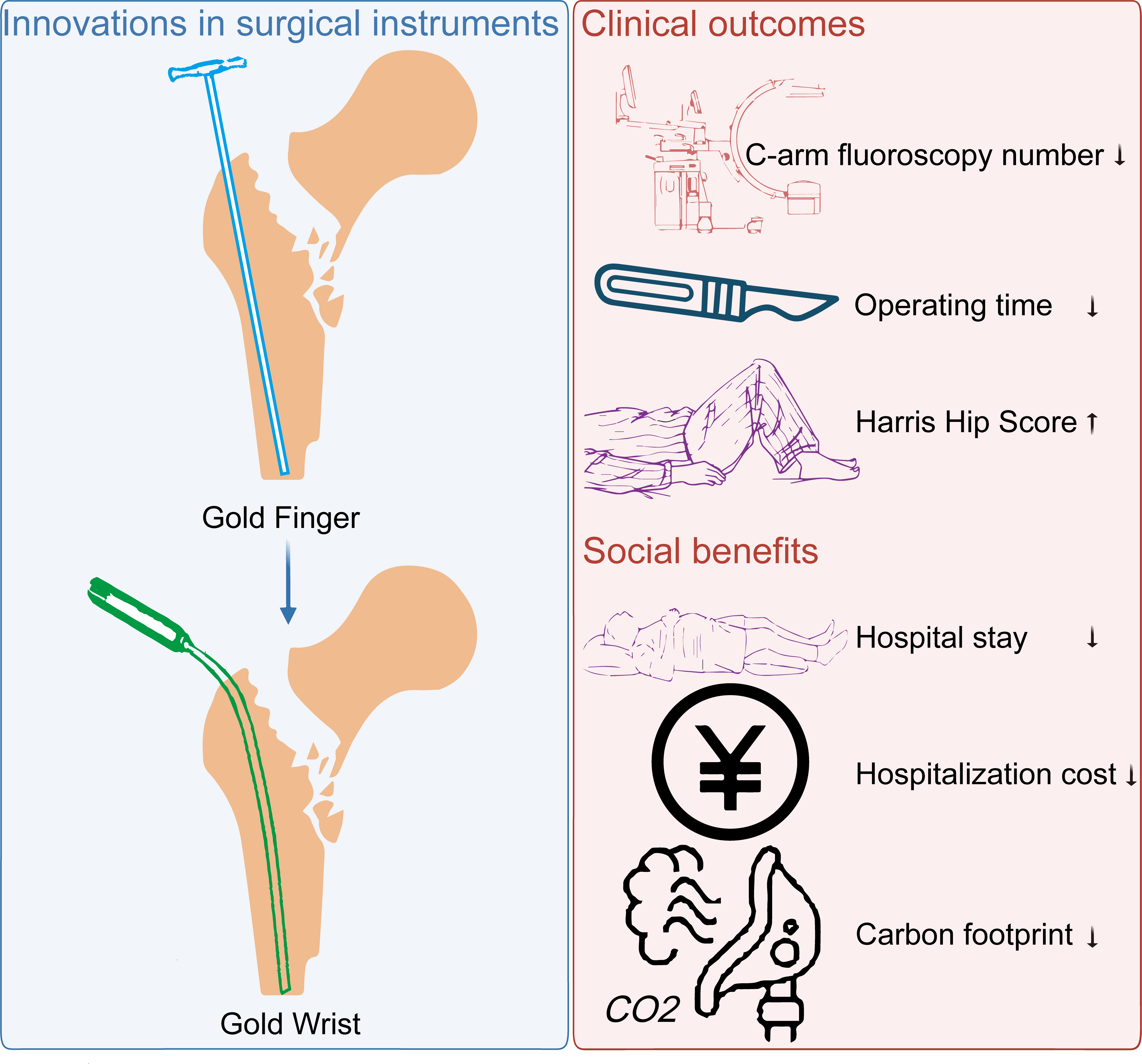

Supplement: S1 Fig — (TIF) [file pone.0348432.s001.tif]
